# Supplementary material for: Human Ovarian Reserve from Conception to the Menopause
Source: PLoS One. 2010 Jan 27;5(1):e8772. doi: 10.1371/journal.pone.0008772 (PMC2811725; doi:10.1371/journal.pone.0008772)
Supplement: File S1 — This file contains (1) a plot of the ADC model that best fits the histological data, (2) full statistics for the model, (3) a plot of the residuals, (4) the dataset with values for the model, the residuals, the 95% CI for the model and the 95% prediction interval, (5) details of the iterations taken to obtain the model, and (6) the ranking of the peak functions as fitted to the dataset. (0.18 MB PDF) [file pone.0008772.s001.pdf]

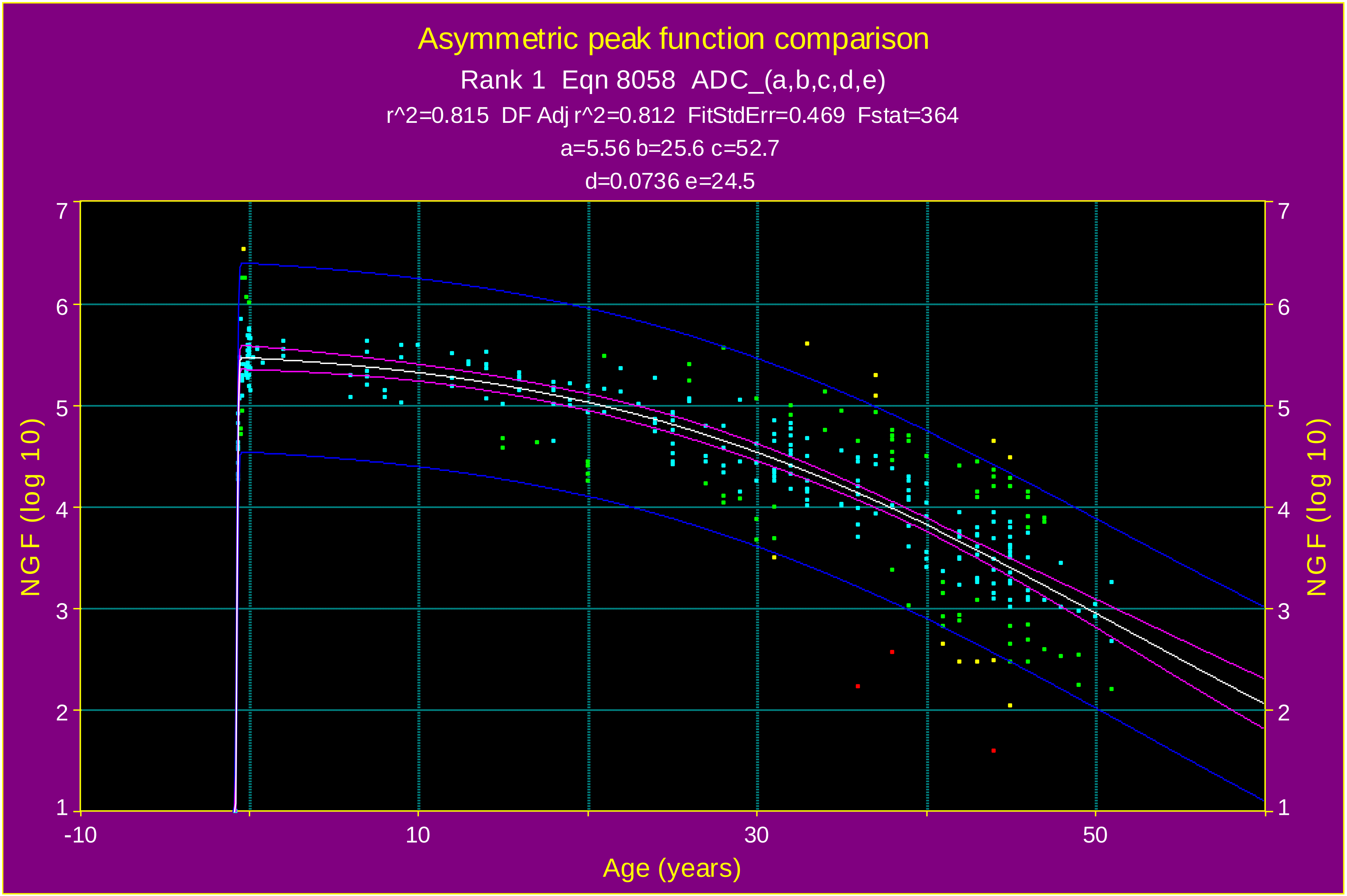
Asymmetric peak function comparison
Rank 1 Eqn 8058 ADC_(a,b,c,d,e)
r^2=0.815 DF Adj r^2=0.812 FitStdErr=0.469 Fstat=364
a=5.56 b=25.6 c=52.7
d=0.0736 e=24.5
NGF (log 10)
Age (years)
-10
10
30
50
1
2
3
4
5
6
7

Rank 1 Eqn 8058 ADC_(a,b,c,d,e)

r^2 Coef Det	DF Adj r^2	Fit Std Err	F-value
0.8149677513	0.8121642324	0.4685812354	364.46933930

Parm	Value	Std Error	t-value	95% Confidence Limits		P>|t|
a	5.564115648	0.091411466	60.86890292	5.384294961	5.743936335	0.00000
b	25.63253000	0.390602477	65.62306057	24.86415368	26.40090631	0.00000
c	52.66953996	0.782200033	67.33512875	51.13082984	54.20825007	0.00000
d	0.073591444	0.005823061	12.63793194	0.062136571	0.085046317	0.00000
e	24.47368229	2.093805220	11.68861462	20.35483912	28.59252547	0.00000

Area Xmin-Xmax	Area Precision		
236.72241175	1.200998e-12		
Function min	X-Value	Function max	X-Value
2.8697690156	51.000000000	5.4739437290	-0.406399719
1st Deriv min	X-Value	1st Deriv max	X-Value
-0.090629102	51.000000000	29.685409287	-0.702257763
2nd Deriv min	X-Value	2nd Deriv max	X-Value
-0.000930544	1.8192307692	-0.000146363	51.000000000

Procedure	Minimization	Iterations	
LevMarqdt	Least Squares	32	
r^2 Coef Det	DF Adj r^2	Fit Std Err	Max Abs Err
0.8149677513	0.8121642324	0.4685812354	1.8957426254
r^2 Attainable			
0.8623295057			

Source	Sum of Squares	DF	Mean Square	F Statistic	P>F
Regr	320.10376	4	80.02594	364.469	0.00000
Error	72.677132	331	0.21956837		
Total	392.78089	335			
Lack Fit	18.602792	71	0.26201116	1.2598	0.10031
Pure Err	54.07434	260	0.20797823		

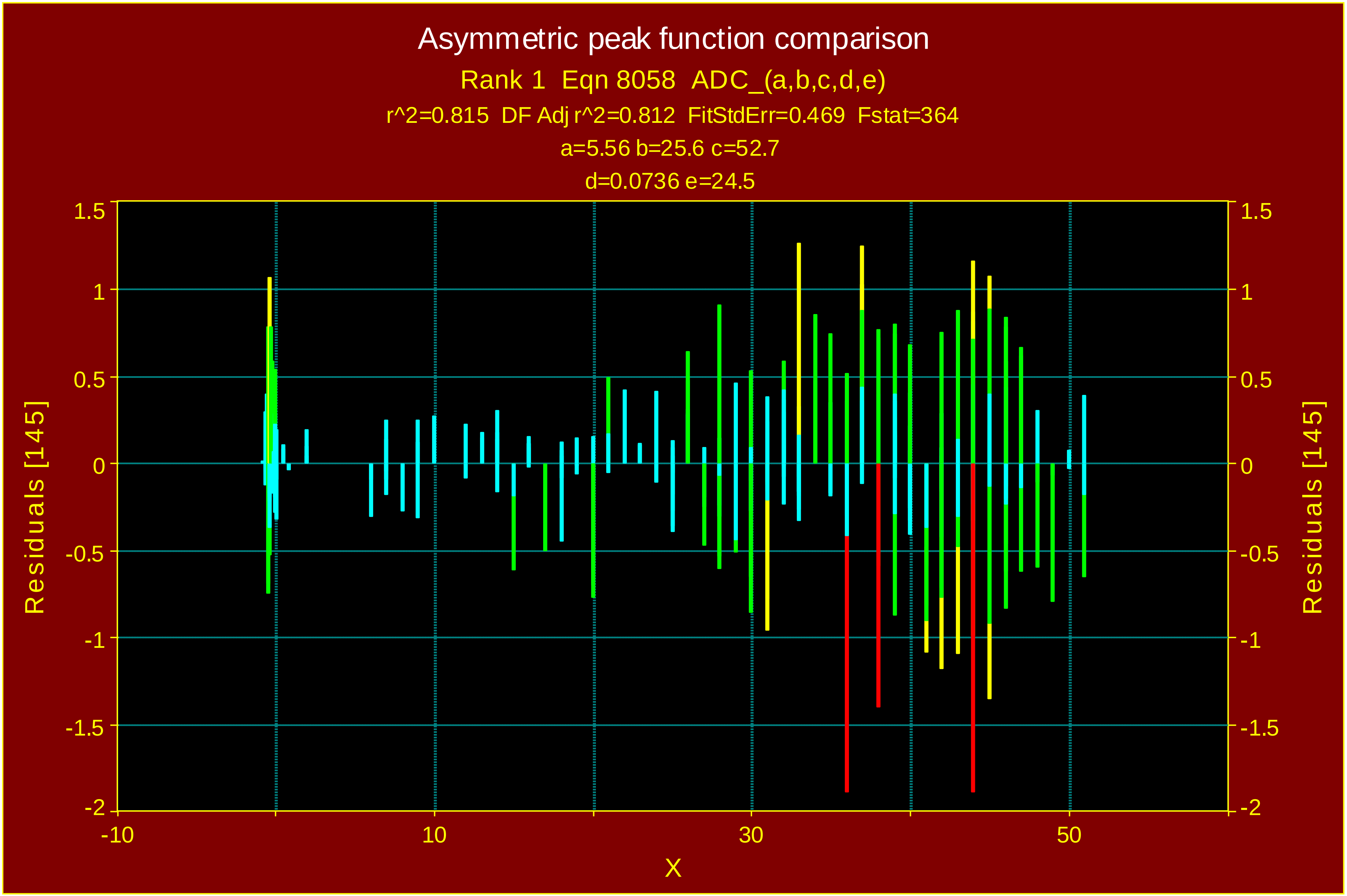

Asymmetric peak function comparison
Rank 1 Eqn 8058 ADC_(a,b,c,d,e)
r^2=0.815 DF Adj r^2=0.812 FitStdErr=0.469 Fstat=364
a=5.56 b=25.6 c=52.7
d=0.0736 e=24.5
Residuals [145]
X
1.5
1
0.5
0
-0.5
-1
-1.5
-2
-10
10
30
50

Rank 1 Eqn 8058 ADC_(a,b,c,d,e)

XY	* X Value	Y Value	Y Predict	Residual	Residual%	95% Confidence Limits		95% Prediction Limits		Weights
1	-0.769231	1.0000000	0.9932161	0.0067839	0.6783875	0.7164350	1.2699973	0.0307854	1.9556468	1
2	-0.769231	1.0000000	0.9932161	0.0067839	0.6783875	0.7164350	1.2699973	0.0307854	1.9556468	1
3	-0.769231	1.0000000	0.9932161	0.0067839	0.6783875	0.7164350	1.2699973	0.0307854	1.9556468	1
4	-0.769231	1.0000000	0.9932161	0.0067839	0.6783875	0.7164350	1.2699973	0.0307854	1.9556468	1
5	-0.769231	1.0000000	0.9932161	0.0067839	0.6783875	0.7164350	1.2699973	0.0307854	1.9556468	1
6	-0.769231	1.0000000	0.9932161	0.0067839	0.6783875	0.7164350	1.2699973	0.0307854	1.9556468	1
7	-0.769231	1.0000000	0.9932161	0.0067839	0.6783875	0.7164350	1.2699973	0.0307854	1.9556468	1
8	-0.769231	1.0000000	0.9932161	0.0067839	0.6783875	0.7164350	1.2699973	0.0307854	1.9556468	1
9	-0.769231	1.0000000	0.9932161	0.0067839	0.6783875	0.7164350	1.2699973	0.0307854	1.9556468	1
10	-0.769231	1.0000000	0.9932161	0.0067839	0.6783875	0.7164350	1.2699973	0.0307854	1.9556468	1
11	-0.769231	1.0000000	0.9932161	0.0067839	0.6783875	0.7164350	1.2699973	0.0307854	1.9556468	1
12	-0.640110	4.3259465	4.3850191	-0.059073	-1.365542	4.0775423	4.6924959	3.4133160	5.3567222	1
13	-0.640110	4.2660199	4.3850191	-0.118999	-2.789466	4.0775423	4.6924959	3.4133160	5.3567222	1
14	-0.637363	4.3103746	4.4412022	-0.130828	-3.035179	4.1377622	4.7446423	3.4707689	5.4116356	1
15	-0.637363	4.6328014	4.4412022	0.1915991	4.1357079	4.1377622	4.7446423	3.4707689	5.4116356	1
16	-0.637363	4.4271127	4.4412022	-0.014090	-0.318256	4.1377622	4.7446423	3.4707689	5.4116356	1
17	-0.626374	4.5935962	4.6476199	-0.054024	-1.176066	4.3637031	4.9315366	3.6831129	5.6121269	1
18	-0.623626	4.5703094	4.6946144	-0.124305	-2.719837	4.4163360	4.9728927	3.7317520	5.6574767	1
19	-0.612637	4.9150303	4.8643464	0.0506839	1.0312014	4.6109543	5.1177386	3.9083796	5.8203133	1
20	-0.609890	4.8269619	4.9023078	-0.075346	-1.560938	4.6555806	5.1490350	3.9480860	5.8565297	1
21	-0.601648	5.0604863	5.0058897	0.0545966	1.0788803	4.7797291	5.2320504	4.0567777	5.9550018	1
22	-0.583333	5.4747988	5.1851070	0.2896918	5.2913695	5.0039815	5.3662325	4.2457075	6.1245065	1
23	-0.582418	5.4041492	5.1923941	0.2117551	3.9183807	5.0133715	5.3714167	4.2533978	6.1313904	1
24	-0.500000	5.8516252	5.4585527	0.3930725	6.7173219	5.3457853	5.5713201	4.5299077	6.3871977	1
25	-0.480769	4.7150335	5.4676200	-0.752587	-15.96142	5.3527976	5.5824423	4.5387232	6.3965167	1
26	-0.442308	4.7742906	5.4733039	-0.699013	-14.64120	5.3561353	5.5904724	4.5441142	6.4024936	1
27	-0.416667	6.2525132	5.4739120	0.7786012	12.452613	5.3563203	5.5915037	4.5446688	6.4031551	1
28	-0.403846	4.9446208	5.4739422	-0.529321	-10.70499	5.3562915	5.5915928	4.5446915	6.4031928	1
29	-0.403846	5.0953019	5.4739422	-0.378640	-7.431165	5.3562915	5.5915928	4.5446915	6.4031928	1
30	-0.384615	5.2458087	5.4738592	-0.228051	-4.347290	5.3562058	5.5915126	4.5446082	6.4031102	1
31	-0.384615	5.2952613	5.4738592	-0.178598	-3.372787	5.3562058	5.5915126	4.5446082	6.4031102	1
32	-0.384615	5.2708255	5.4738592	-0.203034	-3.852028	5.3562058	5.5915126	4.5446082	6.4031102	1
33	-0.333000	6.5334924	5.4734251	1.0600673	16.225125	5.3559270	5.5909232	4.5441938	6.4026564	1
34	-0.288462	5.4009919	5.4730139	-0.072022	-1.333495	5.3556819	5.5903459	4.5438036	6.4022242	1
35	-0.250000	6.2564050	5.4726563	0.7837487	12.527141	5.3554692	5.5898435	4.5434643	6.4018484	1
36	-0.166667	5.3283796	5.4718774	-0.143498	-2.693086	5.3550045	5.5887504	4.5427250	6.4010299	1
37	-0.166667	5.2944662	5.4718774	-0.177411	-3.350880	5.3550045	5.5887504	4.5427250	6.4010299	1
38	-0.166667	6.0564475	5.4718774	0.5845700	9.6520283	5.3550045	5.5887504	4.5427250	6.4010299	1
39	-0.125000	5.3692159	5.4714859	-0.102270	-1.904747	5.3547701	5.5882016	4.5423531	6.4006186	1
40	-0.125000	5.3201463	5.4714859	-0.151340	-2.844651	5.3547701	5.5882016	4.5423531	6.4006186	1

41	-0.115385	5.5332050	5.4713953	0.0618097	1.1170688	5.3547158	5.5880748	4.5422671	6.4005234	1
42	-0.083333	5.4149733	5.4710929	-0.056120	-1.036376	5.3545344	5.5876513	4.5419799	6.4002058	1
43	-0.083333	5.4031205	5.4710929	-0.067972	-1.258020	5.3545344	5.5876513	4.5419799	6.4002058	1
44	-0.038462	5.6824898	5.4706680	0.2118218	3.7276233	5.3542790	5.5870571	4.5415763	6.3997598	1
45	-0.038462	5.5874584	5.4706680	0.1167904	2.0902234	5.3542790	5.5870571	4.5415763	6.3997598	1
46	-0.038462	5.4871030	5.4706680	0.0164350	0.2995201	5.3542790	5.5870571	4.5415763	6.3997598	1
47	-0.038462	5.5383550	5.4706680	0.0676870	1.2221497	5.3542790	5.5870571	4.5415763	6.3997598	1
48	-0.038462	5.2673243	5.4706680	-0.203344	-3.860475	5.3542790	5.5870571	4.5415763	6.3997598	1
49	0.0000000	5.1846914	5.4703026	-0.285611	-5.508740	5.3540589	5.5865463	4.5412290	6.3993761	1
50	0.0000000	5.3636120	5.4703026	-0.106691	-1.989156	5.3540589	5.5865463	4.5412290	6.3993761	1
51	0.0000000	5.3783979	5.4703026	-0.091905	-1.708774	5.3540589	5.5865463	4.5412290	6.3993761	1
52	0.0000000	5.2988531	5.4703026	-0.171450	-3.235596	5.3540589	5.5865463	4.5412290	6.3993761	1
53	0.0000000	5.7458552	5.4703026	0.2755526	4.7956763	5.3540589	5.5865463	4.5412290	6.3993761	1
54	0.0000000	5.5301997	5.4703026	0.0598971	1.0830914	5.3540589	5.5865463	4.5412290	6.3993761	1
55	0.0000000	5.7551123	5.4703026	0.2848097	4.9488120	5.3540589	5.5865463	4.5412290	6.3993761	1
56	0.0000000	6.0050947	5.4703026	0.5347921	8.9056397	5.3540589	5.5865463	4.5412290	6.3993761	1
57	0.0000000	5.4996871	5.4703026	0.0293845	0.5342941	5.3540589	5.5865463	4.5412290	6.3993761	1
58	0.0000000	5.5910646	5.4703026	0.1207620	2.1599112	5.3540589	5.5865463	4.5412290	6.3993761	1
59	0.0000000	5.5831988	5.4703026	0.1128962	2.0220701	5.3540589	5.5865463	4.5412290	6.3993761	1
60	0.0000000	5.6646420	5.4703026	0.1943394	3.4307446	5.3540589	5.5865463	4.5412290	6.3993761	1
61	0.0000000	5.6893089	5.4703026	0.2190063	3.8494356	5.3540589	5.5865463	4.5412290	6.3993761	1
62	0.0000000	5.5403295	5.4703026	0.0700269	1.2639482	5.3540589	5.5865463	4.5412290	6.3993761	1
63	0.0833330	5.3655460	5.4695066	-0.103961	-1.937558	5.3535778	5.5854353	4.5404724	6.3985407	1
64	0.0833330	5.6611025	5.4695066	0.1915959	3.3844274	5.3535778	5.5854353	4.5404724	6.3985407	1
65	0.0833330	5.1426397	5.4695066	-0.326867	-6.356013	5.3535778	5.5854353	4.5404724	6.3985407	1
66	0.2500000	5.4633995	5.4678972	-0.004498	-0.082325	5.3525992	5.5831953	4.5389415	6.3968529	1
67	0.5000000	5.5445740	5.4654393	0.0791347	1.4272453	5.3510887	5.5797899	4.5366008	6.3942779	1
68	0.5000000	5.5693739	5.4654393	0.1039346	1.8661805	5.3510887	5.5797899	4.5366008	6.3942779	1
69	0.8333333	5.4197906	5.4620790	-0.042288	-0.780260	5.3489936	5.5751644	4.5333954	6.3907627	1
70	2.0000000	5.5465427	5.4495399	0.0970027	1.7488865	5.3408837	5.5581962	4.5213852	6.3776947	1
71	2.0000000	5.4888820	5.4495399	0.0393420	0.7167584	5.3408837	5.5581962	4.5213852	6.3776947	1
72	2.0000000	5.6378648	5.4495399	0.1883249	3.3403584	5.3408837	5.5581962	4.5213852	6.3776947	1
73	6.0000000	5.2922561	5.3962208	-0.103965	-1.964468	5.3020218	5.4904197	4.4696472	6.3227943	1
74	6.0000000	5.0827854	5.3962208	-0.313435	-6.166607	5.3020218	5.4904197	4.4696472	6.3227943	1
75	7.0000000	5.2833012	5.3800693	-0.096768	-1.831584	5.2890731	5.4710655	4.4538159	6.3063227	1
76	7.0000000	5.3283796	5.3800693	-0.051690	-0.970083	5.2890731	5.4710655	4.4538159	6.3063227	1
77	7.0000000	5.5185139	5.3800693	0.1384446	2.5087302	5.2890731	5.4710655	4.4538159	6.3063227	1
78	7.0000000	5.6283889	5.3800693	0.2483196	4.4119129	5.2890731	5.4710655	4.4538159	6.3063227	1
79	7.0000000	5.1947918	5.3800693	-0.185278	-3.566602	5.2890731	5.4710655	4.4538159	6.3063227	1
80	8.0000000	5.1398791	5.3626588	-0.222780	-4.334338	5.2745987	5.4507189	4.4366892	6.2886283	1
81	8.0000000	5.1397249	5.3626588	-0.222934	-4.337468	5.2745987	5.4507189	4.4366892	6.2886283	1
82	8.0000000	5.0791812	5.3626588	-0.283478	-5.581166	5.2745987	5.4507189	4.4366892	6.2886283	1

83	9.0000000	5.0218258	5.3439224	-0.322097	-6.413933	5.2584804	5.4293644	4.4181981	6.2696466	1
84	9.0000000	5.5932861	5.3439224	0.2493637	4.4582685	5.2584804	5.4293644	4.4181981	6.2696466	1
85	9.0000000	5.4653829	5.3439224	0.1214605	2.2223602	5.2584804	5.4293644	4.4181981	6.2696466	1
86	10.000000	5.5943926	5.3237926	0.2705999	4.8369845	5.2406033	5.4069820	4.3982736	6.2493117	1
87	12.000000	5.5041744	5.2790835	0.2250910	4.0894595	5.1991562	5.3590107	4.3538519	6.2043150	1
88	12.000000	5.1875207	5.2790835	-0.091563	-1.765058	5.1991562	5.3590107	4.3538519	6.2043150	1
89	12.000000	5.2718416	5.2790835	-0.007242	-0.137368	5.1991562	5.3590107	4.3538519	6.2043150	1
90	13.000000	5.3961993	5.2543697	0.1418296	2.6283249	5.1754105	5.3333289	4.3292213	6.1795181	1
91	13.000000	5.4281348	5.2543697	0.1737651	3.2011935	5.1754105	5.3333289	4.3292213	6.1795181	1
92	14.000000	5.5289167	5.2279949	0.3009218	5.4426901	5.1495616	5.3064282	4.3028912	6.1530986	1
93	14.000000	5.4031205	5.2279949	0.1751256	3.2411941	5.1495616	5.3064282	4.3028912	6.1530986	1
94	14.000000	5.3638056	5.2279949	0.1358107	2.5319837	5.1495616	5.3064282	4.3028912	6.1530986	1
95	14.000000	5.0594820	5.2279949	-0.168513	-3.330635	5.1495616	5.3064282	4.3028912	6.1530986	1
96	15.000000	4.6720979	5.1998944	-0.527797	-11.29678	5.1215677	5.2782210	4.2747997	6.1249890	1
97	15.000000	4.5797836	5.1998944	-0.620111	-13.54018	5.1215677	5.2782210	4.2747997	6.1249890	1
98	15.000000	5.0058495	5.1998944	-0.194045	-3.876363	5.1215677	5.2782210	4.2747997	6.1249890	1
99	16.000000	5.1405143	5.1700051	-0.029491	-0.573693	5.0914063	5.2486039	4.2448874	6.0951228	1
100	16.000000	5.3201047	5.1700051	0.1500996	2.8213660	5.0914063	5.2486039	4.2448874	6.0951228	1
101	16.000000	5.2833012	5.1700051	0.1132961	2.1444192	5.0914063	5.2486039	4.2448874	6.0951228	1
102	16.000000	5.2815560	5.1700051	0.1115509	2.1120845	5.0914063	5.2486039	4.2448874	6.0951228	1
103	16.000000	5.2600714	5.1700051	0.0900663	1.7122636	5.0914063	5.2486039	4.2448874	6.0951228	1
104	17.000000	4.6323763	5.1382663	-0.505890	-10.92074	5.0590723	5.2174603	4.2130978	6.0634348	1
105	18.000000	5.2236556	5.1046197	0.1190359	2.2787849	5.0245749	5.1846645	4.1793780	6.0298614	1
106	18.000000	5.1760913	5.1046197	0.0714716	1.3808021	5.0245749	5.1846645	4.1793780	6.0298614	1
107	18.000000	5.0128372	5.1046197	-0.091782	-1.830948	5.0245749	5.1846645	4.1793780	6.0298614	1
108	18.000000	5.1461280	5.1046197	0.0415084	0.8065938	5.0245749	5.1846645	4.1793780	6.0298614	1
109	18.000000	4.6532125	5.1046197	-0.451407	-9.700979	5.0245749	5.1846645	4.1793780	6.0298614	1
110	19.000000	5.0000000	5.0690101	-0.069010	-1.380202	4.9879341	5.1500861	4.1436786	5.9943416	1
111	19.000000	5.2087664	5.0690101	0.1397563	2.6830980	4.9879341	5.1500861	4.1436786	5.9943416	1
112	19.000000	5.0530784	5.0690101	-0.015932	-0.315286	4.9879341	5.1500861	4.1436786	5.9943416	1
113	20.000000	4.2552725	5.0313859	-0.776113	-18.23886	4.9491772	5.1135946	4.1059545	5.9568173	1
114	20.000000	4.3222193	5.0313859	-0.709167	-16.40746	4.9491772	5.1135946	4.1059545	5.9568173	1
115	20.000000	5.1847510	5.0313859	0.1533651	2.9580039	4.9491772	5.1135946	4.1059545	5.9568173	1
116	20.000000	4.9258121	5.0313859	-0.105574	-2.143277	4.9491772	5.1135946	4.1059545	5.9568173	1
117	20.000000	4.3979400	5.0313859	-0.633446	-14.40324	4.9491772	5.1135946	4.1059545	5.9568173	1
118	20.000000	4.4471580	5.0313859	-0.584228	-13.13711	4.9491772	5.1135946	4.1059545	5.9568173	1
119	21.000000	5.4834011	4.9916994	0.4917018	8.9670943	4.9083357	5.0750631	4.0661647	5.9172341	1
120	21.000000	4.9267102	4.9916994	-0.064989	-1.319119	4.9083357	5.0750631	4.0661647	5.9172341	1
121	21.000000	5.1605616	4.9916994	0.1688622	3.2721668	4.9083357	5.0750631	4.0661647	5.9172341	1
122	22.000000	5.1366381	4.9499073	0.1867308	3.6352727	4.8654428	5.0343718	4.0242728	5.8755419	1
123	22.000000	5.3652539	4.9499073	0.4153466	7.7414154	4.8654428	5.0343718	4.0242728	5.8755419	1
124	23.000000	5.0086002	4.9059715	0.1026287	2.0490498	4.8205322	4.9914107	3.9802475	5.8316954	1

125	23.000000	5.0170333	4.9059715	0.1110619	2.2136963	4.8205322	4.9914107	3.9802475	5.8316954	1
126	24.000000	4.8573325	4.8598588	-0.002526	-0.052011	4.7736363	4.9460813	3.9340622	5.7856554	1
127	24.000000	4.7402521	4.8598588	-0.119607	-2.523214	4.7736363	4.9460813	3.9340622	5.7856554	1
128	24.000000	5.2686996	4.8598588	0.4088408	7.7598049	4.7736363	4.9460813	3.9340622	5.7856554	1
129	24.000000	4.8260748	4.8598588	-0.033784	-0.700031	4.7736363	4.9460813	3.9340622	5.7856554	1
130	25.000000	4.8500000	4.8115423	0.0384577	0.7929423	4.7247858	4.8982988	3.8856958	5.7373888	1
131	25.000000	4.4471580	4.8115423	-0.364384	-8.193643	4.7247858	4.8982988	3.8856958	5.7373888	1
132	25.000000	4.4149733	4.8115423	-0.396569	-8.982363	4.7247858	4.8982988	3.8856958	5.7373888	1
133	25.000000	4.6232493	4.8115423	-0.188293	-4.072742	4.7247858	4.8982988	3.8856958	5.7373888	1
134	25.000000	4.9359454	4.8115423	0.1244031	2.5203494	4.7247858	4.8982988	3.8856958	5.7373888	1
135	25.000000	4.5314789	4.8115423	-0.280063	-6.180397	4.7247858	4.8982988	3.8856958	5.7373888	1
136	25.000000	4.7500000	4.8115423	-0.061542	-1.295627	4.7247858	4.8982988	3.8856958	5.7373888	1
137	25.000000	4.9000000	4.8115423	0.0884577	1.8052592	4.7247858	4.8982988	3.8856958	5.7373888	1
138	26.000000	5.0718820	4.7610010	0.3108810	6.1294999	4.6740090	4.8479930	3.8351324	5.6868696	1
139	26.000000	5.0413927	4.7610010	0.2803917	5.5617902	4.6740090	4.8479930	3.8351324	5.6868696	1
140	26.000000	5.0465576	4.7610010	0.2855566	5.6584431	4.6740090	4.8479930	3.8351324	5.6868696	1
141	26.000000	5.2375287	4.7610010	0.4765277	9.0983303	4.6740090	4.8479930	3.8351324	5.6868696	1
142	26.000000	5.4000000	4.7610010	0.6389990	11.833315	4.6740090	4.8479930	3.8351324	5.6868696	1
143	27.000000	4.8000000	4.7082207	0.0917793	1.9120694	4.6213309	4.7951104	3.7823617	5.6340797	1
144	27.000000	4.4471580	4.7082207	-0.261063	-5.870325	4.6213309	4.7951104	3.7823617	5.6340797	1
145	27.000000	4.5051500	4.7082207	-0.203071	-4.507523	4.6213309	4.7951104	3.7823617	5.6340797	1
146	27.000000	4.2296946	4.7082207	-0.478526	-11.31349	4.6213309	4.7951104	3.7823617	5.6340797	1
147	28.000000	4.1139434	4.6531940	-0.539251	-13.10788	4.5667728	4.7396153	3.7273789	5.5790092	1
148	28.000000	4.4000000	4.6531940	-0.253194	-5.754410	4.5667728	4.7396153	3.7273789	5.5790092	1
149	28.000000	4.8000000	4.6531940	0.1468060	3.0584574	4.5667728	4.7396153	3.7273789	5.5790092	1
150	28.000000	4.3424227	4.6531940	-0.310771	-7.156636	4.5667728	4.7396153	3.7273789	5.5790092	1
151	28.000000	5.5586978	4.6531940	0.9055037	16.289854	4.5667728	4.7396153	3.7273789	5.5790092	1
152	28.000000	4.0413927	4.6531940	-0.611801	-15.13838	4.5667728	4.7396153	3.7273789	5.5790092	1
153	28.000000	4.5797836	4.6531940	-0.073410	-1.602924	4.5667728	4.7396153	3.7273789	5.5790092	1
154	29.000000	4.0791812	4.5959212	-0.516740	-12.66774	4.5103511	4.6814913	3.6701851	5.5216573	1
155	29.000000	4.4500000	4.5959212	-0.145921	-3.279128	4.5103511	4.6814913	3.6701851	5.5216573	1
156	29.000000	4.1461280	4.5959212	-0.449793	-10.84851	4.5103511	4.6814913	3.6701851	5.5216573	1
157	29.000000	5.0516811	4.5959212	0.4557599	9.0219445	4.5103511	4.6814913	3.6701851	5.5216573	1
158	30.000000	5.0684901	4.5364099	0.5320802	10.497805	4.4520756	4.6207442	3.6107873	5.4620326	1
159	30.000000	4.4300000	4.5364099	-0.106410	-2.402030	4.4520756	4.6207442	3.6107873	5.4620326	1
160	30.000000	3.8808136	4.5364099	-0.655596	-16.89327	4.4520756	4.6207442	3.6107873	5.4620326	1
161	30.000000	4.6223177	4.5364099	0.0859078	1.8585428	4.4520756	4.6207442	3.6107873	5.4620326	1
162	30.000000	4.2552725	4.5364099	-0.281137	-6.606801	4.4520756	4.6207442	3.6107873	5.4620326	1
163	30.000000	3.6723288	4.5364099	-0.864081	-23.52951	4.4520756	4.6207442	3.6107873	5.4620326	1
164	31.000000	3.6901961	4.4746758	-0.784480	-21.25848	4.3919476	4.5574039	3.5491981	5.4001535	1
165	31.000000	4.3010300	4.4746758	-0.173646	-4.037307	4.3919476	4.5574039	3.5491981	5.4001535	1
166	31.000000	4.7084719	4.4746758	0.2337961	4.9654349	4.3919476	4.5574039	3.5491981	5.4001535	1

167	31.000000	4.0000000	4.4746758	-0.474676	-11.86689	4.3919476	4.5574039	3.5491981	5.4001535	1
168	31.000000	4.8500000	4.4746758	0.3753242	7.7386436	4.3919476	4.5574039	3.5491981	5.4001535	1
169	31.000000	4.3424227	4.4746758	-0.132253	-3.045606	4.3919476	4.5574039	3.5491981	5.4001535	1
170	31.000000	4.3617278	4.4746758	-0.112948	-2.589523	4.3919476	4.5574039	3.5491981	5.4001535	1
171	31.000000	4.6500000	4.4746758	0.1753242	3.7704132	4.3919476	4.5574039	3.5491981	5.4001535	1
172	31.000000	3.5051500	4.4746758	-0.969526	-27.66004	4.3919476	4.5574039	3.5491981	5.4001535	1
173	31.000000	4.2552725	4.4746758	-0.219403	-5.156034	4.3919476	4.5574039	3.5491981	5.4001535	1
174	32.000000	4.9951985	4.4107426	0.5844559	11.700353	4.3299569	4.4915284	3.4854365	5.3360487	1
175	32.000000	4.5563025	4.4107426	0.1455599	3.1946929	4.3299569	4.4915284	3.4854365	5.3360487	1
176	32.000000	4.5119368	4.4107426	0.1011942	2.2428102	4.3299569	4.4915284	3.4854365	5.3360487	1
177	32.000000	4.1723110	4.4107426	-0.238432	-5.714619	4.3299569	4.4915284	3.4854365	5.3360487	1
178	32.000000	4.6020600	4.4107426	0.1913174	4.1572114	4.3299569	4.4915284	3.4854365	5.3360487	1
179	32.000000	4.9000000	4.4107426	0.4892574	9.9848443	4.3299569	4.4915284	3.4854365	5.3360487	1
180	32.000000	4.3222193	4.4107426	-0.088523	-2.048099	4.3299569	4.4915284	3.4854365	5.3360487	1
181	32.000000	4.3979400	4.4107426	-0.012803	-0.291105	4.3299569	4.4915284	3.4854365	5.3360487	1
182	32.000000	4.8260748	4.4107426	0.4153322	8.6060036	4.3299569	4.4915284	3.4854365	5.3360487	1
183	32.000000	4.7000000	4.4107426	0.2892574	6.1544121	4.3299569	4.4915284	3.4854365	5.3360487	1
184	32.000000	4.7708520	4.4107426	0.3601094	7.5481147	4.3299569	4.4915284	3.4854365	5.3360487	1
185	33.000000	4.1461280	4.3446425	-0.198515	-4.787949	4.2660776	4.4232075	3.4195277	5.2697574	1
186	33.000000	4.6686095	4.3446425	0.3239670	6.9392606	4.2660776	4.4232075	3.4195277	5.2697574	1
187	33.000000	5.6042455	4.3446425	1.2596030	22.475870	4.2660776	4.4232075	3.4195277	5.2697574	1
188	33.000000	4.0098756	4.3446425	-0.334767	-8.348561	4.2660776	4.4232075	3.4195277	5.2697574	1
189	33.000000	4.1760913	4.3446425	-0.168551	-4.036102	4.2660776	4.4232075	3.4195277	5.2697574	1
190	33.000000	4.0683158	4.3446425	-0.276327	-6.792167	4.2660776	4.4232075	3.4195277	5.2697574	1
191	33.000000	4.2584936	4.3446425	-0.086149	-2.022991	4.2660776	4.4232075	3.4195277	5.2697574	1
192	33.000000	4.1760913	4.3446425	-0.168551	-4.036102	4.2660776	4.4232075	3.4195277	5.2697574	1
193	33.000000	4.5000000	4.3446425	0.1553575	3.4523878	4.2660776	4.4232075	3.4195277	5.2697574	1
194	33.000000	4.1760913	4.3446425	-0.168551	-4.036102	4.2660776	4.4232075	3.4195277	5.2697574	1
195	34.000000	5.1276166	4.2764161	0.8512005	16.600315	4.2002636	4.3525686	3.3515030	5.2013292	1
196	34.000000	4.7500000	4.2764161	0.4735839	9.9701878	4.2002636	4.3525686	3.3515030	5.2013292	1
197	35.000000	4.5500000	4.2061123	0.3438877	7.5579719	4.1324423	4.2797823	3.2814003	5.1308243	1
198	35.000000	4.9500000	4.2061123	0.7438877	15.028035	4.1324423	4.2797823	3.2814003	5.1308243	1
199	35.000000	4.0127529	4.2061123	-0.193359	-4.818622	4.1324423	4.2797823	3.2814003	5.1308243	1
200	35.000000	4.0233965	4.2061123	-0.182716	-4.541331	4.1324423	4.2797823	3.2814003	5.1308243	1
201	36.000000	2.2380461	4.1337887	-1.895743	-84.70525	4.0625090	4.2050684	3.2092641	5.0583134	1
202	36.000000	4.4500000	4.1337887	0.3162113	7.1058713	4.0625090	4.2050684	3.2092641	5.0583134	1
203	36.000000	4.4900990	4.1337887	0.3563103	7.9354659	4.0625090	4.2050684	3.2092641	5.0583134	1
204	36.000000	4.2013015	4.1337887	0.0675128	1.6069493	4.0625090	4.2050684	3.2092641	5.0583134	1
205	36.000000	4.2500000	4.1337887	0.1162113	2.7343829	4.0625090	4.2050684	3.2092641	5.0583134	1
206	36.000000	3.7075702	4.1337887	-0.426219	-11.49590	4.0625090	4.2050684	3.2092641	5.0583134	1
207	36.000000	4.1267157	4.1337887	-0.007073	-0.171396	4.0625090	4.2050684	3.2092641	5.0583134	1
208	36.000000	4.6500000	4.1337887	0.5162113	11.101318	4.0625090	4.2050684	3.2092641	5.0583134	1

209	36.000000	3.8300752	4.1337887	-0.303714	-7.929702	4.0625090	4.2050684	3.2092641	5.0583134	1
210	36.000000	3.9912261	4.1337887	-0.142563	-3.571901	4.0625090	4.2050684	3.2092641	5.0583134	1
211	37.000000	5.0863598	4.0595115	1.0268483	20.188275	3.9903233	4.1286997	3.1351458	4.9838772	1
212	37.000000	5.3000000	4.0595115	1.2404885	23.405443	3.9903233	4.1286997	3.1351458	4.9838772	1
213	37.000000	4.9344985	4.0595115	0.8749870	17.732034	3.9903233	4.1286997	3.1351458	4.9838772	1
214	37.000000	4.4175215	4.0595115	0.3580100	8.1043185	3.9903233	4.1286997	3.1351458	4.9838772	1
215	37.000000	4.4968743	4.0595115	0.4373628	9.7259294	3.9903233	4.1286997	3.1351458	4.9838772	1
216	37.000000	3.9340184	4.0595115	-0.125493	-3.189946	3.9903233	4.1286997	3.1351458	4.9838772	1
217	38.000000	4.6558776	3.9833551	0.6725226	14.444593	3.9157116	4.0509985	3.0591036	4.9076065	1
218	38.000000	4.5440680	3.9833551	0.5607130	12.339450	3.9157116	4.0509985	3.0591036	4.9076065	1
219	38.000000	4.0103848	3.9833551	0.0270297	0.6739931	3.9157116	4.0509985	3.0591036	4.9076065	1
220	38.000000	3.3859636	3.9833551	-0.597391	-17.64318	3.9157116	4.0509985	3.0591036	4.9076065	1
221	38.000000	4.7500000	3.9833551	0.7666449	16.139894	3.9157116	4.0509985	3.0591036	4.9076065	1
222	38.000000	4.4591210	3.9833551	0.4757659	10.669500	3.9157116	4.0509985	3.0591036	4.9076065	1
223	38.000000	4.3802112	3.9833551	0.3968562	9.0602065	3.9157116	4.0509985	3.0591036	4.9076065	1
224	38.000000	2.5751878	3.9833551	-1.408167	-54.68212	3.9157116	4.0509985	3.0591036	4.9076065	1
225	38.000000	4.7000000	3.9833551	0.7166449	15.247765	3.9157116	4.0509985	3.0591036	4.9076065	1
226	39.000000	3.0246909	3.9054021	-0.880711	-29.11740	3.8384820	3.9723221	2.9812033	4.8296008	1
227	39.000000	4.1630122	3.9054021	0.2576101	6.1880703	3.8384820	3.9723221	2.9812033	4.8296008	1
228	39.000000	3.8160092	3.9054021	-0.089393	-2.342576	3.8384820	3.9723221	2.9812033	4.8296008	1
229	39.000000	4.0917724	3.9054021	0.1863704	4.5547585	3.8384820	3.9723221	2.9812033	4.8296008	1
230	39.000000	4.6500000	3.9054021	0.7445979	16.012858	3.8384820	3.9723221	2.9812033	4.8296008	1
231	39.000000	4.0606978	3.9054021	0.1552958	3.8243612	3.8384820	3.9723221	2.9812033	4.8296008	1
232	39.000000	4.7000000	3.9054021	0.7945979	16.906339	3.8384820	3.9723221	2.9812033	4.8296008	1
233	39.000000	4.3002693	3.9054021	0.3948672	9.1823836	3.8384820	3.9723221	2.9812033	4.8296008	1
234	39.000000	3.6070259	3.9054021	-0.298376	-8.272084	3.8384820	3.9723221	2.9812033	4.8296008	1
235	39.000000	4.2628069	3.9054021	0.3574048	8.3842604	3.8384820	3.9723221	2.9812033	4.8296008	1
236	40.000000	3.4087486	3.8257433	-0.416995	-12.23307	3.7584554	3.8930312	2.9015178	4.7499688	1
237	40.000000	3.5563025	3.8257433	-0.269441	-7.576431	3.7584554	3.8930312	2.9015178	4.7499688	1
238	40.000000	4.0413927	3.8257433	0.2156494	5.3360165	3.7584554	3.8930312	2.9015178	4.7499688	1
239	40.000000	3.9000000	3.8257433	0.0742567	1.9040178	3.7584554	3.8930312	2.9015178	4.7499688	1
240	40.000000	3.4913617	3.8257433	-0.334382	-9.577398	3.7584554	3.8930312	2.9015178	4.7499688	1
241	40.000000	4.2304489	3.8257433	0.4047056	9.5664934	3.7584554	3.8930312	2.9015178	4.7499688	1
242	40.000000	4.5000000	3.8257433	0.6742567	14.983482	3.7584554	3.8930312	2.9015178	4.7499688	1
243	41.000000	2.9196010	3.7444772	-0.824876	-28.25304	3.6755084	3.8134459	2.8201278	4.6688265	1
244	41.000000	3.1500000	3.7444772	-0.594477	-18.87229	3.6755084	3.8134459	2.8201278	4.6688265	1
245	41.000000	2.6532125	3.7444772	-1.091265	-41.12994	3.6755084	3.8134459	2.8201278	4.6688265	1
246	41.000000	2.8325089	3.7444772	-0.911968	-32.19648	3.6755084	3.8134459	2.8201278	4.6688265	1
247	41.000000	3.2595939	3.7444772	-0.484883	-14.87557	3.6755084	3.8134459	2.8201278	4.6688265	1
248	41.000000	3.3698650	3.7444772	-0.374612	-11.11653	3.6755084	3.8134459	2.8201278	4.6688265	1
249	42.000000	3.4891144	3.6617095	-0.172595	-4.946674	3.5896150	3.7338039	2.7371217	4.5862973	1
250	42.000000	3.5051500	3.6617095	-0.156559	-4.466556	3.5896150	3.7338039	2.7371217	4.5862973	1

251	42.000000	2.4771213	3.6617095	-1.184588	-47.82116	3.5896150	3.7338039	2.7371217	4.5862973	1
252	42.000000	2.9410142	3.6617095	-0.720695	-24.50499	3.5896150	3.7338039	2.7371217	4.5862973	1
253	42.000000	3.2304489	3.6617095	-0.431261	-13.34986	3.5896150	3.7338039	2.7371217	4.5862973	1
254	42.000000	2.8873359	3.6617095	-0.774374	-26.81966	3.5896150	3.7338039	2.7371217	4.5862973	1
255	42.000000	3.7000000	3.6617095	0.0382905	1.0348791	3.5896150	3.7338039	2.7371217	4.5862973	1
256	42.000000	3.7500000	3.6617095	0.0882905	2.3544141	3.5896150	3.7338039	2.7371217	4.5862973	1
257	42.000000	3.7529699	3.6617095	0.0912604	2.4316847	3.5896150	3.7338039	2.7371217	4.5862973	1
258	42.000000	3.9458623	3.6617095	0.2841529	7.2012865	3.5896150	3.7338039	2.7371217	4.5862973	1
259	42.000000	4.4067275	3.6617095	0.7450180	16.906378	3.5896150	3.7338039	2.7371217	4.5862973	1
260	43.000000	3.8000000	3.5775531	0.2224469	5.8538650	3.5008652	3.6542411	2.6525958	4.5025105	1
261	43.000000	4.0883488	3.5775531	0.5107956	12.493935	3.5008652	3.6542411	2.6525958	4.5025105	1
262	43.000000	3.7323938	3.5775531	0.1548406	4.1485609	3.5008652	3.6542411	2.6525958	4.5025105	1
263	43.000000	3.2952371	3.5775531	-0.282316	-8.567396	3.5008652	3.6542411	2.6525958	4.5025105	1
264	43.000000	2.4771213	3.5775531	-1.100432	-44.42382	3.5008652	3.6542411	2.6525958	4.5025105	1
265	43.000000	3.0877814	3.5775531	-0.489772	-15.86161	3.5008652	3.6542411	2.6525958	4.5025105	1
266	43.000000	4.1500000	3.5775531	0.5724469	13.793900	3.5008652	3.6542411	2.6525958	4.5025105	1
267	43.000000	3.2787536	3.5775531	-0.298800	-9.113205	3.5008652	3.6542411	2.6525958	4.5025105	1
268	43.000000	4.4500000	3.5775531	0.8724469	19.605548	3.5008652	3.6542411	2.6525958	4.5025105	1
269	43.000000	3.2648178	3.5775531	-0.312735	-9.578951	3.5008652	3.6542411	2.6525958	4.5025105	1
270	43.000000	3.5215957	3.5775531	-0.055957	-1.588980	3.5008652	3.6542411	2.6525958	4.5025105	1
271	43.000000	3.7160033	3.5775531	0.1384502	3.7257828	3.5008652	3.6542411	2.6525958	4.5025105	1
272	43.000000	3.6127839	3.5775531	0.0352307	0.9751684	3.5008652	3.6542411	2.6525958	4.5025105	1
273	44.000000	3.6948683	3.4921275	0.2027408	5.4870908	3.4094536	3.5748015	2.5666547	4.4176004	1
274	44.000000	3.3856063	3.4921275	-0.106521	-3.146298	3.4094536	3.5748015	2.5666547	4.4176004	1
275	44.000000	4.3576300	3.4921275	0.8655024	19.861770	3.4094536	3.5748015	2.5666547	4.4176004	1
276	44.000000	3.1518293	3.4921275	-0.340298	-10.79685	3.4094536	3.5748015	2.5666547	4.4176004	1
277	44.000000	3.1000000	3.4921275	-0.392128	-12.64928	3.4094536	3.5748015	2.5666547	4.4176004	1
278	44.000000	4.3000000	3.4921275	0.8078725	18.787731	3.4094536	3.5748015	2.5666547	4.4176004	1
279	44.000000	4.6500000	3.4921275	1.1578725	24.900483	3.4094536	3.5748015	2.5666547	4.4176004	1
280	44.000000	2.4913617	3.4921275	-1.000766	-40.16943	3.4094536	3.5748015	2.5666547	4.4176004	1
281	44.000000	3.8549737	3.4921275	0.3628461	9.4124151	3.4094536	3.5748015	2.5666547	4.4176004	1
282	44.000000	3.9460099	3.4921275	0.4538823	11.502311	3.4094536	3.5748015	2.5666547	4.4176004	1
283	44.000000	3.4827307	3.4921275	-0.009397	-0.269813	3.4094536	3.5748015	2.5666547	4.4176004	1
284	44.000000	4.2000000	3.4921275	0.7078725	16.854106	3.4094536	3.5748015	2.5666547	4.4176004	1
285	44.000000	3.2500000	3.4921275	-0.242128	-7.450078	3.4094536	3.5748015	2.5666547	4.4176004	1
286	44.000000	1.6020600	3.4921275	-1.890068	-117.9773	3.4094536	3.5748015	2.5666547	4.4176004	1
287	45.000000	3.6989700	3.4055582	0.2934118	7.9322565	3.3156479	3.4954686	2.4794109	4.3317056	1
288	45.000000	4.4801507	3.4055582	1.0745925	23.985633	3.3156479	3.4954686	2.4794109	4.3317056	1
289	45.000000	2.6565773	3.4055582	-0.748981	-28.19346	3.3156479	3.4954686	2.4794109	4.3317056	1
290	45.000000	2.8305887	3.4055582	-0.574970	-20.31272	3.3156479	3.4954686	2.4794109	4.3317056	1
291	45.000000	3.0172421	3.4055582	-0.388316	-12.86990	3.3156479	3.4954686	2.4794109	4.3317056	1
292	45.000000	3.0860037	3.4055582	-0.319555	-10.35496	3.3156479	3.4954686	2.4794109	4.3317056	1

293	45.000000	4.2898453	3.4055582	0.8842870	20.613495	3.3156479	3.4954686	2.4794109	4.3317056	1
294	45.000000	3.8500000	3.4055582	0.4444418	11.543942	3.3156479	3.4954686	2.4794109	4.3317056	1
295	45.000000	3.5483894	3.4055582	0.1428312	4.0252404	3.3156479	3.4954686	2.4794109	4.3317056	1
296	45.000000	3.5185139	3.4055582	0.1129557	3.2103248	3.3156479	3.4954686	2.4794109	4.3317056	1
297	45.000000	3.5962671	3.4055582	0.1907089	5.3029685	3.3156479	3.4954686	2.4794109	4.3317056	1
298	45.000000	3.3500000	3.4055582	-0.055558	-1.658454	3.3156479	3.4954686	2.4794109	4.3317056	1
299	45.000000	4.2000000	3.4055582	0.7944418	18.915281	3.3156479	3.4954686	2.4794109	4.3317056	1
300	45.000000	4.2000000	3.4055582	0.7944418	18.915281	3.3156479	3.4954686	2.4794109	4.3317056	1
301	45.000000	2.0472749	3.4055582	-1.358283	-66.34592	3.3156479	3.4954686	2.4794109	4.3317056	1
302	45.000000	3.5000000	3.4055582	0.0944418	2.6983367	3.3156479	3.4954686	2.4794109	4.3317056	1
303	45.000000	3.2500000	3.4055582	-0.155558	-4.786407	3.3156479	3.4954686	2.4794109	4.3317056	1
304	45.000000	3.8000000	3.4055582	0.3944418	10.380047	3.3156479	3.4954686	2.4794109	4.3317056	1
305	45.000000	3.6289506	3.4055582	0.2233924	6.1558396	3.3156479	3.4954686	2.4794109	4.3317056	1
306	45.000000	2.4771213	3.4055582	-0.928437	-37.48048	3.3156479	3.4954686	2.4794109	4.3317056	1
307	45.000000	3.2652896	3.4055582	-0.140269	-4.295747	3.3156479	3.4954686	2.4794109	4.3317056	1
308	45.000000	3.6188323	3.4055582	0.2132740	5.8934493	3.3156479	3.4954686	2.4794109	4.3317056	1
309	46.000000	4.1000000	3.3179761	0.7820239	19.073753	3.2197536	3.4161987	2.3909849	4.2449674	1
310	46.000000	2.6937269	3.3179761	-0.624249	-23.17418	3.2197536	3.4161987	2.3909849	4.2449674	1
311	46.000000	3.9000000	3.3179761	0.5820239	14.923689	3.2197536	3.4161987	2.3909849	4.2449674	1
312	46.000000	3.9000000	3.3179761	0.5820239	14.923689	3.2197536	3.4161987	2.3909849	4.2449674	1
313	46.000000	2.4771213	3.3179761	-0.840855	-33.94484	3.2197536	3.4161987	2.3909849	4.2449674	1
314	46.000000	2.8450980	3.3179761	-0.472878	-16.62080	3.2197536	3.4161987	2.3909849	4.2449674	1
315	46.000000	3.1760913	3.3179761	-0.141885	-4.467280	3.2197536	3.4161987	2.3909849	4.2449674	1
316	46.000000	4.1500000	3.3179761	0.8320239	20.048768	3.2197536	3.4161987	2.3909849	4.2449674	1
317	46.000000	3.0791812	3.3179761	-0.238795	-7.755143	3.2197536	3.4161987	2.3909849	4.2449674	1
318	46.000000	3.1075491	3.3179761	-0.210427	-6.771478	3.2197536	3.4161987	2.3909849	4.2449674	1
319	46.000000	3.5035864	3.3179761	0.1856103	5.2977223	3.2197536	3.4161987	2.3909849	4.2449674	1
320	46.000000	3.7500000	3.3179761	0.4320239	11.520636	3.2197536	3.4161987	2.3909849	4.2449674	1
321	46.000000	3.8000000	3.3179761	0.4820239	12.684838	3.2197536	3.4161987	2.3909849	4.2449674	1
322	47.000000	3.8880671	3.2295173	0.6585498	16.937718	3.1220880	3.3369466	2.3015054	4.1575292	1
323	47.000000	2.6020600	3.2295173	-0.627457	-24.11387	3.1220880	3.3369466	2.3015054	4.1575292	1
324	47.000000	3.8450980	3.2295173	0.6155808	16.009495	3.1220880	3.3369466	2.3015054	4.1575292	1
325	47.000000	3.0827854	3.2295173	-0.146732	-4.759718	3.1220880	3.3369466	2.3015054	4.1575292	1
326	48.000000	3.0224284	3.1403219	-0.117893	-3.900621	3.0229647	3.2576790	2.2111084	4.0695354	1
327	48.000000	3.4424798	3.1403219	0.3021579	8.7773331	3.0229647	3.2576790	2.2111084	4.0695354	1
328	48.000000	2.5340261	3.1403219	-0.606296	-23.92618	3.0229647	3.2576790	2.2111084	4.0695354	1
329	49.000000	2.2479733	3.0505338	-0.802560	-35.70151	2.9226875	3.1783800	2.1199373	3.9811302	1
330	49.000000	2.9698816	3.0505338	-0.080652	-2.715667	2.9226875	3.1783800	2.1199373	3.9811302	1
331	49.000000	2.5440680	3.0505338	-0.506466	-19.90771	2.9226875	3.1783800	2.1199373	3.9811302	1
332	50.000000	3.0364293	2.9602998	0.0761295	2.5072038	2.8215480	3.0990516	2.0281426	3.8924570	1
333	50.000000	2.9247960	2.9602998	-0.035504	-1.213890	2.8215480	3.0990516	2.0281426	3.8924570	1
334	51.000000	2.2108534	2.8697690	-0.658916	-29.80368	2.7198269	3.0197111	1.9358806	3.8036575	1

335	51.000000	3.2552725	2.8697690	0.3855035	11.842434	2.7198269	3.0197111	1.9358806	3.8036575	1
336	51.000000	2.6848454	2.8697690	-0.184924	-6.887684	2.7198269	3.0197111	1.9358806	3.8036575	1

Rank 1 Eqn 8058 ADC_(a,b,c,d,e)

Precision	Error @Xmin	Error @Xmean	Error @Xmax
18	1.199786e-15	5.476343e-19	0.0000000000
17	-5.68989e-15	-1.29607e-17	-1.23919e-17
16	6.334407e-14	-1.00491e-16	-6.91376e-17
15	3.596879e-13	-7.77641e-17	-2.51842e-16
14	3.517949e-13	-2.38677e-15	-5.01493e-16
13	-1.37969e-10	2.537053e-14	1.097163e-13
12	-9.28381e-10	-3.07716e-13	-1.19841e-13
11	-7.82778e-09	1.065801e-11	2.115554e-11
10	-6.69835e-08	1.180588e-10	2.007756e-10
9	3.275691e-07	5.812223e-10	1.179352e-09
8	3.214315e-07	-4.94134e-09	-7.43234e-09
7	3.283909e-07	9.003848e-08	6.695328e-08
6	0.0001977365	3.009804e-08	-8.1815e-07
5	-0.004744533	1.190841e-06	1.889804e-05
4	0.0554136915	-3.47409e-06	-8.88319e-05
3	1.1871345333	-0.001232687	-0.001317829
2	-0.999236404	0.0177908476	0.0243120372

1	0.8149677513	105	**8058**	ADC_(a,b,c,d,e)
2	0.8114857718	73	**8166**	GMG_(a,b,c,d)
3	0.7678769051	53	**8066**	PulseWid2_(a,b,c,d)
4	0.7678769051	23	**8068**	IntermedPk_(a,b,c,d)
5	0.7678451318	42	**8070**	EquilPk_(a,b,c,d,e)
6	0.7663722546	27	**8062**	LgstcPowPk_(a,b,c,d)
7	0.7660176955	75	**8164**	EMG_(a,b,c,d)
8	0.7645280231	62	**8182**	ChiSq_(a,b,c,d)
9	0.7644709523	57	**8064**	PulsePow_(a,b,c,d)
10	0.7529455641	34	**8050**	Gamma_(a,b,c,d)
11	0.7349939061	31	**8174**	LogNorm4_(a,b,c,d)
12	0.7009778927	76	**8052**	Weibull_(a,b,c,d)
13	0.6545237561	70	**8186**	Pearson IV_(a,b,c,d,e)
14	0.6480059927	97	**8184**	FVar_(a,b,c,d,e)
15	0.3764707236	49	**8180**	InvGamma_(a,b,c,d)
16	0.3662982779	16	**8036**	Pulse_(a,b,c)
17	0.3591277391	24	**8033**	ExtrVal_(a,b,c)
18	0.3530441312	32	**8178**	ExtrVal4F_(a,b,c,d)
19	0.2219458490	79	**8054**	Beta_(a,b,c,d,e)
20	0.2210226821	33	**8056**	ADS_(a,b,c,d,e)
