# Supplementary material for: Human Ovarian Reserve from Conception to the Menopause
Source: PLoS One. 2010 Jan 27;5(1):e8772. doi: 10.1371/journal.pone.0008772 (PMC2811725; doi:10.1371/journal.pone.0008772)
Supplement: File S2 — It may have been a chance result that the ADC performed well on the 325 datapoints selected, and that another model would perform better, in general, on similar datasets. The additional file contains details of the testing of this hypothesis, and shows that the ADC mean r2 was statistically significantly higher than any other peak model (p = 0:0065). (0.05 MB PDF) [file pone.0008772.s002.pdf]

Model robustness analysis.

It is possible that the ADC model is highest ranked for the total dataset, but that some other model would be higher ranked in general for similar datasets to our combined histological data. In order to check this, the peak function models were ranked 61 times on datasets consisting of the original 325 points minus 50 points chosen at random. Two models were consistently highly ranked: the ADC and GMG models. The mean r^2 coefficients obtained over the multiple runs were compared for a statistically significant difference. The results are given in the table below.

Model ID	Runs	Mean r^2	Std Dev of r^2	Min. r^2	Max. r^2
8058-ADC	61	0.805	0.014	0.773	0.824
8166-GMG	61	0.796	0.021	0.738	0.821
8066	61	0.762	0.010	0.747	0.783
8068	61	0.762	0.010	0.747	0.783
8062	61	0.761	0.010	0.745	0.781
8164	61	0.760	0.010	0.745	0.781
8182	61	0.756	0.009	0.741	0.773
8184	61	0.752	0.010	0.721	0.771
8070	61	0.747	0.092	0.224	0.783
8064	61	0.740	0.101	0.343	0.796
8174	61	0.730	0.013	0.674	0.752
8052	61	0.699	0.080	0.497	0.776
8050	61	0.688	0.037	0.599	0.737
8186	61	0.651	0.011	0.635	0.673
8180	61	0.360	0.011	0.337	0.379
8036	61	0.341	0.009	0.325	0.362
8033	61	0.332	0.010	0.316	0.353
8178	61	0.324	0.010	0.308	0.346
8054	61	0.305	0.266	0.017	0.734
8056	61	0.179	0.028	0.074	0.223

T-test analysis of difference of the two highest-ranked means:
ADC model mean: 0.805 (sd=0.014) (se=0.0018)
GMG model mean: 0.796 (sd=0.021) (se=0.0027)

Difference between means:
0.009 (sd=0.033) (se=0.0032)
95% CI: 0.0027<diff<0.0153 (Wald)

t-value of difference: 2.785; df-t: 104
Probability: 0.996754 (left tail pr: 0.00325)
Doublesided p-value: 0.0065
